# The Preventive Effects of Greenshell Mussel (*Perna canaliculus)* on Early-Stage Metabolic Osteoarthritis in Rats with Diet-Induced Obesity

**DOI:** 10.3390/nu11071601

**Published:** 2019-07-15

**Authors:** Parkpoom Siriarchavatana, Marlena C. Kruger, Matthew R. Miller, Hong Sabrina Tian, Frances M. Wolber

**Affiliations:** 1 School of Food and Advanced Technology, Massey University, Palmerston North 4442, New Zealand; 2 School of Health Sciences, Massey University, Palmerston North 4442, New Zealand; 3 Riddet Centre of Research Excellence, Massey University, Palmerston North 4442, New Zealand; 4 Cawthron Institute, Nelson 7042, New Zealand; 5 Sanford Ltd., Auckland 1010, New Zealand; 6 Centre for Metabolic Health Research, Massey University, Palmerston North 4442, New Zealand

**Keywords:** osteoarthritis, metabolic dysregulation, obesity, diet-induced obese rats, Greenshell mussel, *Perna canaliculus*

## Abstract

The prevalence of osteoarthritis (OA) is rising worldwide, with the most pronounced increase being in the category of metabolic-associated osteoarthritis (MetOA). This is predicted to worsen with the global rise in aging societies and obesity. To address this health burden, research is being conducted to identify foods that can reduce the incidence or severity of MetOA. Oil from the Greenshell mussel (*Perna canaliculus*) (GSM), a native New Zealand shellfish, has been successfully used to reduce OA symptoms. The current study assessed the effect of including flash-dried powder from whole GSM meat as part of a normal (control) versus high-fat/high-sugar (HFHS) diet for 13 weeks on the development of MetOA in rats. Rats fed a HFHS diet developed metabolic dysregulation and obesity with elevated plasma leptin and HbA1C concentrations. Visible damage to knee joint cartilage was minimal, but plasma levels of C telopeptide of type II collagen (CTX-II), a biomarker of cartilage degradation, were markedly higher in HFHS-fed rats compared to control-fed rats. However, rats fed the HFHS diet containing GSM had significantly reduced serum CTX-II. Inclusion of GSM in rats fed the control diet also lowered CTX-II. These findings suggest that dietary GSM can reduce the incidence or slow the progression of early MetOA.

## 1. Introduction

Osteoarthritis (OA) is a chronic disease of joints featuring articular cartilage erosion resulting in progressive pain and joint immobility. It affects 3.7% of the global population, equating to approximately 268 million people [1]. The prevalence increases by age group. OA can be classified into four subtypes based on its pathogenesis: post-traumatic, genetic predisposing, ageing, and metabolic-associated osteoarthritis (MetOA). It is projected that MetOA incidence will continue to increase in parallel with the incidence of obesity. 

The relationship between obesity and osteoarthritis is not due simply to the physical demands caused by additional body; this is evident because frequently osteoarthritis in obese patients occurs in non-weight bearing joints such as the fingers, hands, wrists and temporomandibular joint [2]. Instead, systemic factors associated with low-grade chronic inflammation are implicated. In animals fed high-energy diets, this is indicated by the presence of inflammatory cells, especially macrophages, in adipose tissue [3]. Obese rats display a significant increase both in the volume of white adipose tissue in the body and in the size of individual adipocytes. Adipocytes are capable of producing many types of proinflammatory cytokines and chemokines such as tumor necrosis factor-α (TNF-α), interleukin 1 (IL-1), interleukin 6 (IL-6), interferon-γ IFN-γ, macrophage inflammatory protein-1 (MIP-1), growth-related oncogene-α (GRO-α), and regulated on activation, normal T cell expressed and secreted (RANTES) [4]. Moreover, they secrete adipokines into the blood circulation. One of the most significant adipokines is leptin, which has an influence on chronic inflammation [5] and the development of osteoarthritis due to obesity [6,7,8].

The greenlipped or Greenshell™ mussel (GSM) (*Perna canaliculus*) is an important commercial marine species of New Zealand. Its lipid fractions contain high amounts of long-chain omega-3 polyunsaturated fatty acids (eicosapentaenoic acid (20:5n-3 EPA) and docosahexaenoic (22:6n-3 DHA)) as well as various types of interesting minor fatty acids: 5, 9, 12, 15-octadecatetraenoic acid (C18:4); 5, 9, 12, 16-nonadecatetraenoic acid (C19:4); 7, 11, 14, 17-eicosatetraenoic acid (C20:4); and 5, 9, 12, 15, 18-heneicosapentaenoic acid (C21:5) [9]. Long-chain omega-3 polyunsaturated fatty acids such as EPA and DHA compounds are known to have anti-inflammatory properties. In particular, the GSM oil fraction extracted by supercritical fluidic CO_2_ method has been shown to have an inhibitory effect on prostaglandinE2 (PGE2) in lipopolysaccharide (LPS)-activated mononuclear cells [10] via both the cyclooxygenase pathway [11] and the lipoxygenase pathway [9]. Its molecular mechanisms have been studied in LPS-induced RAW 264.7 cells and found to involve the NF-κB and MAPK kinase signaling pathways [12].

In a study employing an adjuvant-induced arthritis rat model, a GSM oil extract of 15 mg/kg body weight had more potent anti-inflammatory effects than doses of 25 mg naproxen, 50 mg ibuprofen, 300 mg aspirin or 300 mg dried mussel, respectively [10]. This mussel oil at a dose of 300 mg/kg caused no gastrointestinal toxicity, a common side effect of aspirin. It also had an analgesic effect at the initial phase of inflammation; ex vivo splenocytes reduced production of the pro-inflammatory cytokines, TNF-α and IFN-γ, but increased the anti-inflammatory cytokine IL-10 [13]. Pre-clinical veterinary studies involved with GSM have also been recently reviewed. Most of these studies were predicated on the hypothesis that anti-inflammatory activity of mussel lipids would be due to long-chain polyunsaturated fatty acids, omega-3 (LCPUFA_s_) or furan fatty acid [14]. Although there remains strong evidence supporting the health benefits of mussel oil in arthritis via regulation of inflammatory processes, animal model studies published to date have been carried out only in post-traumatic osteoarthritis models by using chemical or surgical induction to destabilize the joints. The current study was designed to focus specifically on MetOA, which is a chronic disorder and the most debilitating affliction in the OA population.

## 2. Materials and Methods 

### 2.1. Analysis of GSM Composition

Flash-dried powder from whole GSM meat was produced by Sanford Ltd (ENZAQ facility, Blenheim, New Zealand) using standard manufacturing processes and assessed for proximate composition in a commercial testing laboratory (Food Testing Laboratory of Cawthron Analytical Services; Nelson, New Zealand). The Association of Official Analytical Chemists (AOAC) methods for crude protein (AOAC 981.10), total fat (AOAC 948.15), moisture at 105 °C (AOAC 950.46) and ash (AOAC 920.153) were used and carbohydrate content was determined by calculation (100% − % crude protein − % total fat − % moisture − % ash). An aliquot of the total lipid extract from the GSM powder was trans-methylated in methanol/chloroform/hydrochloric acid (10:1:1, v/v/v) for 1 h at 100 °C. After the addition of water, the mixture was extracted three times with hexane/chloroform (4:1, v/v) to obtain fatty acid methyl esters (FAME). Samples were made up to 1 mL with an internal injection standard (19:0 FAME) and analyzed by gas chromatography mass spectrometry (GC-MS) according to AOAC 963.22.

### 2.2. Animal Study

Forty-eight female Sprague Dawley (SD) rats aged 11 weeks reared on standard chow were obtained from the Small Animal Production Unit at Massey University (Palmerston North, New Zealand) and the study was conducted in the same animal facility as approved by the Massey University Animal Ethics Committee (MUAEC protocol/approval 16/112). The animal room environment was set at 22 ± 1 °C, with 45%–55% humidity and a 12/12 light-dark cycle throughout the study. The rats were singly housed in conventional cages with heat-treated aspen wood shavings as bedding. After a one-week acclimatization period, the rats were randomized into test groups and fed one of four diets (Specialty Feeds, Glen Forrest, Western Australia) for 13 weeks:

(1) normal control diet (ND) containing 5% total fat (from soy oil), 5% sucrose, and 15% total protein (from casein);

(2) normal control diet supplemented with GSM (ND + GSM) containing 5% total fat (84% from soy oil/16% from GSM), 5% sucrose, and 15% total protein (66% from casein/33% from GSM);

(3) high-fat high-sugar diet (HFHS) containing 30% total fat (50% from soy oil/50% from lard), 30% sucrose, and 15% total protein (from casein);

(4) high-fat high-sugar diet supplemented with GSM (HFHS + GSM) containing 30% total fat (49% from soy oil/49% from lard/1% from GSM), 30% sucrose, and 15% total protein (66% from casein/33% from GSM).

Fresh food was provided daily and the previous day’s food intake was measured. At the end of the study, rats were food-deprived overnight. All rats were anesthetized and subjected to dual energy X-ray absorptiometry scanning (DXA) as described elsewhere [15] to measure lean mass and fat mass. Plasma was collected from EDTA-anticoagulated blood samples drawn by cardiac puncture and stored at −80 °C until analysis. Retroperitoneal, epididymal, inguinal, and interscapular fat pads were individually dissected and weighed. Knee joints were dissected out and stored in formalin prior to further processing as described below.

### 2.3. Plasma Analysis

Duo-set kits for rat IL10, TNF-alfa, IL-6, IL1β, Quantikine Mouse/Rat leptin and Quantikine Rat total adiponectin/Acrp30 kits were obtained from R&D System, Minneapolis, USA. CTX2 assay kit was obtained from Cloud-Clone Corp, TX, USA. HbA1C assay kit was obtained from (Fine Test, Wuhan Fine Biological Technology, Wuhan, China). All assays were carried out following the manufacturer’s instructions and the optical density was measured using a microplate reader (Multiskan FC, Thermo Fisher Scientific, Vantaa, Finland).

### 2.4. Histopathological Assessment

After the knee joints were dissected, each of them was separately submersed in 10% buffer formalin fixative followed by a decalcification process in 10% EDTA buffer for four weeks. Knee joints were cut along the coronal plane at the middle of the collateral ligament and embedded in paraffin. Two sections were cut at a thickness of 3 µm and stained with safranin-O. Articular cartilage at the tibial plateau was evaluated by 14-point Mankin score criteria [16], which evaluates the formality of structure, cellularity, matrix staining, and tidemark.

### 2.5. Data Analysis

Statistical analysis was performed using IBM statistics software version 25 (Armonk, NY, USA). Data were analyzed by one-way ANOVA or Student’s t-test. Multiple comparisons were analyzed by the least significant difference test, and knee joint scoring was done by the Mann–Whitney U test.

## 3. Results

### 3.1. GSM Composition

The GSM powder used to supplement the rat diets was comprised of approximately 43% protein, 22% carbohydrate, 21% ash, 8% fat, and 6% moisture (Table 1). The fatty acids were equally represented by saturated and polyunsaturated fatty acids, with monounsaturated fatty acids being present at much lower levels.

### 3.2. Animal Study

After 13 weeks on the test diets, HFHD-fed rats had significantly higher body weight gain than ND-fed rats, as expected. Body composition data revealed the weight gain was due to additional fat deposition in the body, as percent body fat was almost double in HFHS-fed rats compared with ND-fed rats. Retroperitoneal and epididymal fat masses rose from approximately 10 g each in ND-fed rats to nearly 20 g in HFHS-fed rats, corresponding with the approximate doubling in percent total body fat. The inguinal fat pad, although smaller, also showed a near-doubling between ND and HFHS rats, with GSM inclusion in the HFHS diet resulting in a significant increase. Only the interscapular fat pad showed a minor difference in weight between the diet groups, although the gain was statistically significant in the HFHS + GSM group (Table 2).

**Figure 1:**
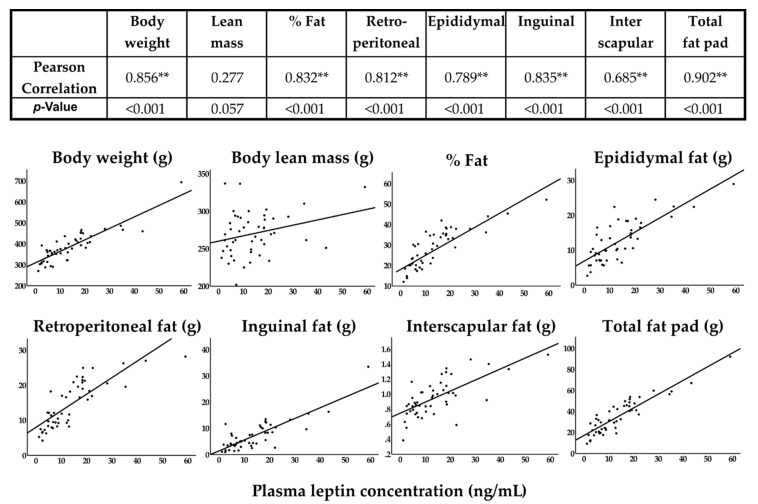
Correlation of plasma leptin with body composition and fat pads was evaluated by the Pearson correlation method. Asterisks indicate a significant difference. “Total fat pads” is the summation of retroperitoneal, epididymal, inguinal, and interscapular fat pad. Scatter plot charts represent the correlation of plasma leptin with each individual parameter.

Plasma analytes are shown in Table 3. Plasma cytokines were detected at low (picogram/mL) concentrations with high variability between subjects, and showed no trends or statistical differences between test groups. HbA1C (glycated hemoglobin), a biomarker for high blood glucose, was elevated in HFHS-fed rats, with a significant difference in the HFHS + GSM group when compared to ND-fed rats. Leptin was highly variable between individuals; however, leptin levels in rats fed either of the NDs were <9000 pg/mL while the HFHS diets resulted in more than double those levels. Adiponectin was present in very high (µg/mL) concentrations but did not differ between groups. Only leptin was highly correlated with body weight, percent body fat, and specific fat pads; leptin was not correlated with lean mass (Figure 1).

**Figure 2:**
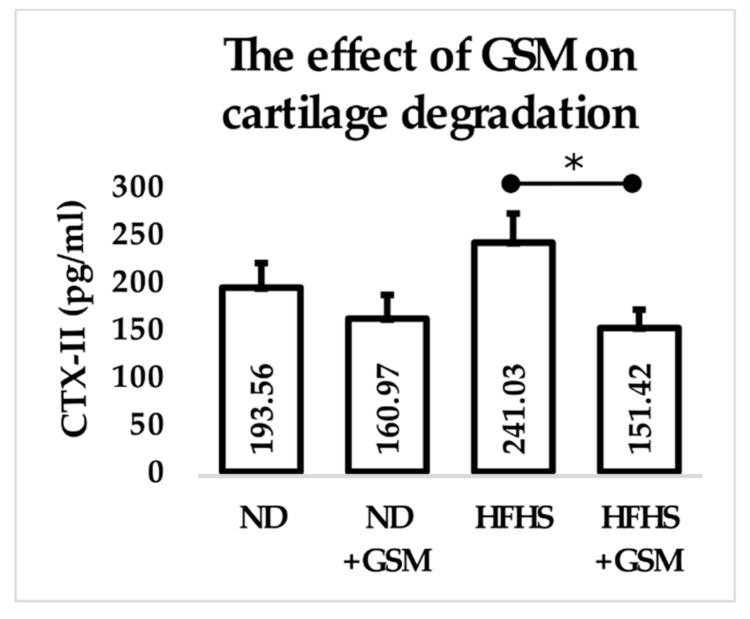
Modulation of cartilage degradation by diet. Plasma samples were assessed in duplicate for C-terminus telopeptide of type II collagen (CTX-II) by competitive ELISA. Data are shown as mean + standard deviation of *n* = 11–12 rats per group, and the mean values inserted into each bar. * *p* ≤ 0.05 between the groups within a diet by Student’s *t*-test.

To evaluate the presence of early osteoarthritis in the rats’ joints, cartilage degradation was measured using the plasma biomarker C-terminus telopeptide of type II collagen (CTX-II). The results showed that rats fed the HFHS diet had a higher level of CTX-II than rats fed either of the NDs. Interestingly, including GSM in the diet slightly reduced CTX-II in the ND rats and significantly reduced CTX-II in the HFHS rats (Figure 2).

**Figure 3:**
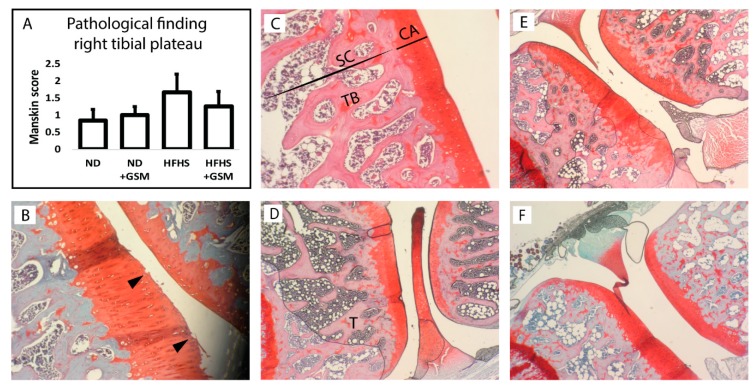
(**A**): Histopathological assessment of osteoarthritis in right knee joints: Mankin score was used to measure the severity of osteoarthritis at the tibial plateau. Scores for each histological aspect were summed for each individual. Data are shown as mean + standard error of mean of *n* = 11–12 rats per diet group. No significant difference was detected by analyzing with the Mann–Whitney U test. Knee joint at microscopic levels, 40 and 100× magnification: Articular cartilage of tibia bone (T) from each group, ND (**C**), ND + GSM (**D**), HFHS (**E**), and HFHS + GSM (**F**). Dark red staining represents cartilaginous layers (CA), which have similarity in color and thickness across all groups. Most of the slices have smooth surface cartilage and seem to be normal articular cartilage. Surface irregularities were found in some tissue samples from HFHS as indicated by black arrowheads (**B**). SC = subchondral bone, TB = trabecular bone.

As the CTX-II data indicated the presence of cartilage degradation at least in the HFHS group, the rat knee joints were assessed. The cartilage layers were microscopically examined in accordance with the Mankin scoring system (Figure 3A). All histological sections presented a normal appearance with similar thicknesses of cartilage layers and homogeneous color stains (Figure 3C–F). A small amount of irregularity of articular surface was observed only in the knee joints of rats fed the HFHS diet (Figure 3B) but these were not statistically significant when the Mankin scores were analyzed using a Mann–Whitney U test. However, there was an observable trend for the HFHS rats to have a higher Mankin score than the ND diet rats, and for this to be reduced when GSM was added to the HFHS diet.

## 4. Discussion

Obesity is associated with the progression of OA due not only to increasing weight-loading but also to the many systemic factors produced as part of a chronic inflammatory process. Recent preclinical studies have achieved the establishment of OA in diet-induced animal models without surgical manipulation [17], more closely mimicking the human condition. This animal model represents one particular subtype of human OA, MetOA, and shares with the human both abnormal metabolic markers and pathological changes in articular joints. These markers are associated with reduction of glycemic control, alterations in lipid metabolism, and increases in mediators such as adipokines and cytokines linked with low-grade chronic inflammation [4,7,8,18]. The current study used a similar HFHS diet but assessed MetOA after only 13 weeks rather than 28 weeks. Both HbA1c and CTX-II were significantly elevated, identifying in this model these two biomarkers as being early indicators of MetOA prior to the presence of frank cartilage erosion. Importantly, addition of GSM to the HFHS diet significantly ameliorated CTX-II production, establishing this model and the 13 week time point as being suitable for assessing intervention strategies to prevent or slow the joint pathology.

Variations in outcomes when using this model are likely to depend on the source and proportion of fat in the diet as well as the induction period and the sex of the animals [19,20]. In the current study, diets containing 30% fat and 30% sucrose in diets induced significantly more weight gain than diets containing 5% fat and 5% sucrose. However, the differences in weight gain and mean body weight between diet groups were less than some other published studies. Sun et al. [18] demonstrated accelerated body weight gain, cytokine concentration, and progressive articular cartilage lesions at the joint after 16 weeks of the HFHS diet in male Wistar rats. Collins et al. [17] identified obvious cartilage destruction, severe obesity, and high concentrations of proinflammatory cytokines in both blood and synovial fluid after 28 weeks in male Sprague Dawley rats. Both of these studies used higher sucrose in the diet as well as different sources of fat, with the latter study having a higher concentration of fat in the diet as well. The differences in magnitude in weight gain and other parameters between the current study and previously published studies are likely to be due to testosterone in male rats combined with a higher-energy diet, both of which would influence progression and severity of MetOA [21]. However, using female rats and lowering the fat in the diet slowed the progression of joint disease and allowed for the assessment of preventive effects of GSM powder on the early events of the disorder’s pathogenesis.

Three types of adipocytes with different functionality are situated in various fat depots: brown adipocytes in interscapular fat, beige adipocytes in inguinal fat, and white adipocytes in visceral fat [22]. They produce a range of adipokines, including leptin and adiponectin, which are known to correlate with obesity. White adipocytes release the most leptin into the blood circulation; thus, the amount of visceral fat is highly correlated with plasma leptin concentration [23]. In the current study, the body weight gain in HFHS-fed rats correlated with percent total body fat and in particular with the mass of visceral (epididymal and retroperitoneal) fat, whereas interscapular fat mass was unaffected by diet. Correspondingly, plasma leptin and adiponectin were markedly increased in the HFHS-fed rats. The role of adiponectin and its contribution to the obesity microenvironment has not yet been fully elucidated, as some human and animal studies have shown adiponectin in obesity to be lower than normal [24,25,26,27], suggesting production of adiponectin may be influenced by age, sex, location of excess adipose deposits, or overall health status.

In contrast, consistent evidence indicates that leptin is an initiator for systemic and local inflammation in obesity. Leptin activates toll-like receptor 4 (TLR-4) on innate immune cells such as macrophages and natural killer (NK) cells, resulting in an upregulation of cell proliferation, phagocytosis function, and production of TNF-α, IL-6, IL-1β, and IL-12 [28]. However, the expression of those serum pro-inflammatory cytokines in rats is variable upon many factors [29] and unlikely to have an established reference value. Some studies showed the serum levels of IL-1β, TNF-α, IL-6, and IL-10 in control rats to be around 2–6, 1–11, 0–93, and 6–12 pg/mL respectively [30,31,32]. The result of these cytokines in this study is similar to Collin’s study [17], which showed no significant difference between rats fed a normal diet, and rats fed the HFHS diet. An increase in plasma pro-inflammatory cytokines was not observed in the current study in rats fed a HFHS diet, corresponding with little evidence of synovial damage and thus likely due to the early pathological stage of MetOA.

Inflammatory marker status varies widely and may in fact be minimal or absent in chronic diseases caused by low-grade inflammation such as rheumatoid arthritis, inflammatory bowel disease, atopic dermatitis, psoriasis, and asthma [33]. Aging is also recognized as a low-grade inflammation status. In elderly human subjects, there was no increase in serum inflammatory marker levels but rather an increase in the production of IL-1β, IL-6, and TNF-α in ex vivo culture by mononuclear white blood cells activated with phytohemagglutinin (PHA) plus phorbol myristate acetate (PMA) [34,35]. Thus, low-grade chronic inflammation may not be reflected by changes in serum pro-inflammatory cytokines, but may instead demonstrate changes in discrete microenvironments both in cell function and soluble factors. In the current study, it is reasonable to speculate that low-grade inflammation was successfully established due to leptin upregulation and plasma glucose elevation, which indicate initiation of a metabolic disorder.

Currently, most OA is diagnosed by clinical symptoms and radiographic imaging, at which point significant cartilage damage has already occurred. Early detection of OA is crucial to slow or halt progressive cartilage loss before it becomes debilitating, and this requires early prognostic markers that can both detect early OA and monitor intervention treatment efficacy [36]. C-telopeptide collagen type II (CTX-II) is a metabolic fragment from cartilaginous matrix released by cartilage degradation. It is considered to be one of the most reliable markers for early OA detection and is accurate in predicting further cartilage destruction [37]. It is possible to detect CTX-II in urine as well as serum, and the latter is a commonly used sampling technique in clinical studies as it is less invasive than blood sampling.

The current study showed an increase in plasma CTX-II in HFHS-fed rats but this significantly diminished when the diet was supplemented with GSM. This finding provides further evidence that GSM is protective against OA, and in the absence of changes in inflammatory markers suggests that GSM’s effects may be localized to the cartilage microenvironment. Interestingly, even though the cartilage degradation marker was detected in the blood, the knee joint histopathological findings appeared visually normal. This may be due in part to the fact that CTX-II in the blood circulation represents cartilage degradation events throughout the whole body, whereas histology was conducted on only a single joint. In addition, circulatory CTX-II detection is sensitive and thus measurable levels can likely be identified prior to a cartilage lesion being detectable histologically. A similar finding was observed in a study using a papain-induced osteoarthritis rat model, in which a significant increase of serum CTX-II was detected within a day after inducing papain into the knee joints; CTX-II plateaued at week 2 and remained constant until the study concluded at week 4 [38]. Murat et al. [39] also used the papain model and observed that CTX-II increased within 1 week, but significant changes in Mankin scores in the knee joints did not occur until week 4. Thus, in the rat there is a consensus that CTX-II increases significantly prior to the cartilage lesion becoming explicit.

GSM contains high levels of polyunsaturated fatty acids, especially the omega-3 PUFAs DHA and EPA. The anti-inflammatory [40,41,42,43] and anti-arthritis [44,45,46] effects of omega-3 PUFAs are well established. There is further evidence that omega-3s are important compounds in GSM with potential as anti-inflammatory and anti-arthritis bioactives. The results of the current study validate this and demonstrate the preventive effects of GSM against MetOA in a rat model. However, to date this effect has only been demonstrated in the early stage of MetOA; long-term effects of GSM and its potential ability to treat exacerbated OA in this model are still unknown and should be the subject of further investigation. Such work may reveal additional biomarker changes to confirm ongoing MetOA and identify biomarkers useful for monitoring treatment.

## Figures and Tables

**Table 1:** Nutritive value of Greenshell mussel (GSM) powder and the composition of fatty acids.

**Proximate composition (g/100 g)**	
Fat	8.1
Crude protein	43
Carbohydrate	21.9
Moisture	5.8
Ash	21.2
**Fatty acid profile (% fatty acids)**	
C14:0 myristic acid	5.9
C16:0 palmitic acid	20.3
C17:0 heptadecanoic acid	1.2
C18:0 stearic acid	4.9
C18:1n7 vaccenic acid	3.2
C18:1n9c oleic acid	2.1
C18:2n6c linoleic acid	2.1
C18:3n3 α-linolenic acid (ALA)	1.4
C18:3n4 octadecatrienoic acid	1.3
C18:4n3 stearidonic acid (SDA)	2.5
C20:1 gadoleic acid	2.6
C20:4n6 arachidonic acid (AA)	1.0
C20:5n3 eicosapentaenoic acid (EPA)	13.5
C22:5n3 docosapentaenoic acid (DPA)	1.0
C22:6n3 docosahexaenoic acid (DHA)	10.7
∑SFA	34.29
∑MUFA	8.23
∑PUFA	34.20
∑n-3 PUFA	29.4
∑n-6 PUFA	3.5

∑SFA = sum of saturated fatty acids; ∑MUFA = sum of monounsaturated fatty acids; ∑PUFA = sum of polyunsaturated fatty acids; ∑n-3 PUFA = Omega 3 polyunsaturated fatty acids; ∑n-6 PUFA = Omega 6 polyunsaturated fatty acids.

**Table 2:** Body weight and fat deposition of the rats at the end of the study.

Weight (g)	ND	ND + GSM	HFHS	HFHS + GSM	*p*-Value
BW (week 0)	277.14 ± 8.24	280.47 ± 10.48	280.78 ± 6.53	276.28 ± 9.43	NS
BW (week 13)	343.00 ± 45.70^a^	351.80 ± 51.00^a^	400.80 ± 50.43^b^	417.2 ± 88.08^b^	0.010
% BW gain	24.05 ± 7.95^a^	25.44 ± 8.62^a^	42.42 ± 9.56^b^	48.22 ± 15.72^b^	<0.001
%body fat (week 0)	11.16 ± 3.48	11.36 ± 4.62	11.24 ± 3.64	12.83 ± 4.03	NS
%body fat (week 13)	21.2 ± 5.43^a^	22.88 ± 7.90^a^	34.39 ± 4.80^b^	36.46 ± 7.52^b^	<0.001
%body fat gain	101.13 ± 65.10^a^	112.48 ± 60.96^a^	232.89 ± 98.82^b^	193.91 ± 51.78^b^	<0.001
Lean mass (week 0)	237.68 ± 22.77	236.28 ± 27.05	234.44 ± 20.73	227.39 ± 21.92	NS
Lean mass (week 13)	269.62 ± 32.55	275.29 ± 29.86	266.68 ± 26.49	266.04 ± 29.97	NS
% lean mass gain	13.34 ± 6.52	16.89 ± 8.10	13.77 ± 6.30	17.01 ± 6.54	NS
Retroperitoneal	9.05 ± 3.90^a^	11.14 ± 5.89^a^	19.28 ± 5.01^b^	18.81 ± 5.10^b^	<0.001
Epididymal	8.80 ± 3.04^a^	9.04 ± 3.17^a^	15.31 ± 5.25^b^	17.90 ± 5.73^b^	<0.001
Inguinal	5.23 ± 2.87^a^	4.98 ± 4.42^a^	7.95 ± 3.95^a^	10.71 ± 8.09^b^	0.023
Interscapular	0.83 ± 0.18^a^	0.90 ± 0.23^a^	0.97 ± 0.19^a^	1.10 ± 0.31^b^	0.033

Data are shown as mean ± SD of *n* = 11–12 rats per group and were analyzed by one-way ANOVA or by the least significant difference test for multiple comparison tests. Different superscript letters indicate significant difference at *p* ≤ 0.05. NS = no significance difference, BW = body weight, ND = normal control diet, HFHS = high-fat high-sugar diet.

**Table 3:** Inflammatory and metabolic markers in rat plasma at the end of the study.

Analysts	ND	ND + GSM	HFHS	HFHS + GSM	*p*-Value
IL-1β (pg/mL)	23.90 ± 39.34	4.93 ± 7.45	6.95 ± 15.23	13.46 ± 36.93	NS
IL-6 (pg/mL)	21.90 ± 19.23	29.40 ± 19.67	16.23 ± 22.27	19.56 ± 20.27	NS
IL-10 (pg/mL)	3.36 ± 7.16	7.33 ± 13.11	14.31 ± 38.74	2.39 ± 5.03	NS
TNF-α (pg/mL)	0.16 ± 0.58	0.35 ± 0.81	1.25 ± 3.30	0.00 ± 0.00	NS
HbA1C(ng/mL)	252.25 ± 50.46^a^	227.10 ± 41.59^a^	275.44 ± 67.73^a^	326.76 ± 120.24^b^	0.044
Leptin (ng/mL)	7.71 ± 5.62^a^	8.47 ± 5.52^a^	18.26 ± 10.94^b^	21.72 ± 14.59^b^	0.002
Adiponectin (µg/mL)	8.17 ± 1.97	7.85 ± 2.94	7.92 ± 1.94	9.51 ± 0.96	NS

Rat plasma analytes were measured by ELISA in duplicate wells. Data are shown as mean ± standard deviation and were assessed by one-way ANOVA; NS = no significant difference. Least significant difference method was applied for multiple comparison tests. Different superscript letters indicate significant difference at *p* ≤ 0.05.
